# Toward a macroevolutionary understanding of live‐leaf flammability in plant species of fire‐prone forests

**DOI:** 10.1002/ajb2.70073

**Published:** 2025-07-10

**Authors:** Brad R. Murray, Lyndle K. Hardstaff, Megan L. Murray, Zoe A. Xirocostas

**Affiliations:** ^1^ School of Life Sciences University of Technology Sydney 2007 NSW Australia

**Keywords:** evolution, fire, ignitability, leaf traits, phylogeny, stabilizing selection, wildfire

## Abstract

**Premise:**

The flammability of live leaves in canopies varies considerably among plant species. Identifying macroevolutionary processes that shape variation in leaf flammability contributes to an understanding of the phylogenetic underpinnings of wildfire dynamics.

**Methods:**

We used a phylogenetic comparative approach to examine the macroevolution of live‐leaf flammability in 75 plant species of fire‐prone dry sclerophyll forests in eastern Australia. We estimated phylogenetic signal in leaf flammability, fitted a series of evolutionary models to test macroevolutionary hypotheses about leaf flammability, and assessed evolutionary correlations between leaf flammability and leaf water content (LWC), leaf mass per area (LMA), leaf area (LA), plant growth form, and fire response strategy.

**Results:**

We detected weak phylogenetic signal, indicating that leaf flammability exhibited greater variation among closely related species than would be expected under phylogenetic conservatism. The evolution of leaf flammability was equally well described by an Ornstein–Uhlenbeck model and a Pagel's δ model, implying weak stabilizing selection and an acceleration in leaf flammability evolution over time. We found significant evolutionary correlations such that high leaf flammability was related to low LWC, low LMA, and large LA.

**Conclusions:**

Our results show that live‐leaf flammability is an evolutionarily labile trait in plant species of fire‐prone forests. We suggest that the evolution of the three leaf traits in response to prevailing environmental conditions (such as LWC to water availability, LA to light capture, and LMA to herbivore defence) provide antagonistic selective forces that produce a macroevolutionary pattern of weak stabilising selection on leaf flammability.

Wildfires have influenced the ecology and evolution of plants for millennia (Archibald et al., 2018). Present‐day plant assemblages in fire‐prone regions are composed of species that possess suites of fire‐related survival, reproduction, and flammability‐enhancing traits (Bond and Van Wilgen, 1996; Pausas et al., 2017). Flammability traits that increase a plant's propensity to burn include the retention of dead branches and florets (Schwilk, 2003), the creation of open canopies with fine leaves (Saura‐Mas et al., 2010), and the construction of high‐flammability leaves (Murray et al., 2013). In this context, previous studies have shown considerable interspecific variation in leaf flammability (Engstrom et al., 2004; De Lillis et al., 2009; Ganteaume et al., 2013; Clarke et al., 2014; Grootemaat et al., 2015; Varner et al., 2015; Krix and Murray, 2018). The leaves of some species ignite within seconds (*Acrotriche divaricata*, Ericaceae; Krix et al., 2019), while those of other species can take 10‐fold longer to ignite (*Amyema miquelii*, Loranthaceae; Gill and Moore, 1996). Although phylogenetic relatedness of species and evolutionary correlations between traits can play an important role in shaping phenotypic trait variation in present‐day plant assemblages (Ávila‐Lovera et al., 2023), little is known about the macroevolutionary processes that shape interspecific variation in leaf flammability.

As a result of shared ancestry, phenotypic traits of closely related species are likely to be more similar to each other than to distantly related species (Felsenstein, 1985). Tests of phylogenetic signal, which examine the degree to which traits of related species resemble each other (Blomberg and Garland, 2002), have revealed phylogenetic structure in both leaf and shoot flammability (Simpson et al., 2016; Cui et al., 2020a). For example, New Zealand plant species in the families Ericaceae, Myrtaceae, Pinaceae, and Poaceae have relatively high shoot flammability and species in the Asteraceae have relatively low shoot flammability (Cui et al., 2020b). At the leaf level, Mason et al. (2016) found evidence for phylogenetic signal in leaf ignition temperature across 115 New Zealand plant species. Quantification of phylogenetic signal in a trait is typically measured using Pagel's *λ* (Pagel, 1999) and Blomberg's *K* (Blomberg et al., 2003), both of which are based on a neutral model of trait evolution via random changes over time. These measures of signal provide a key step in unravelling the macroevolution of traits (Münkemüller et al., 2012; Swenson, 2014) and validate evolutionary relatedness among species as a predictor of phenotypic trait variation.

Further insight into the evolution and distribution of phenotypic traits across contemporary species is achieved through the application of mathematical models to test a range of macroevolutionary hypotheses pertinent to patterns throughout a phylogenetic tree, not just among species at the tips of a tree (Pennell and Harmon, 2013; Revell and Harmon, 2022). Exploring macroevolutionary processes is particularly important because different evolutionary processes can produce similar results in terms of phylogenetic signal and different rates of evolution can produce similar estimates of phylogenetic signal (Revell et al., 2008). Previous studies that tested among various Brownian motion (BM) models, including Ornstein–Uhlenbeck (OU) models, have yielded insightful information linking macroevolutionary processes to contemporary distributions of many functional traits across plant species (Agrawal et al., 2009; Flores et al., 2014; Carta et al., 2022; Maccagni and Willi, 2022; Boyko et al., 2023). However, application of phylogenetic comparative methods in this manner has not been applied to flammability traits, despite the potential for such an approach to provide important insight into the evolution of flammability.

In this study, we used a phylogenetic comparative approach to investigate the macroevolution of live‐leaf flammability in plant species inhabiting fire‐prone dry sclerophyll forests across eastern Australia. We studied live‐leaf flammability because leaves on living plants are frequently the first plant structures to ignite and because the capacity of a plant to ignite dictates its contribution to wildfire spread (Gill and Moore, 1996; Murray et al., 2013). We focused on macroevolutionary patterns in the flammability of leaves first and foremost because leaf traits are focal points for selective pressures on plant growth and survival (Reich et al., 1997; Flores et al., 2014). We employed Pagel's *λ* and Blomberg's *K* to test for phylogenetic signal in leaf flammability and then fit and compared a series of BM and OU evolutionary models to test alternative macroevolutionary hypotheses for interspecific variation in leaf flammability across present‐day species. Because traits do not evolve in isolation, we examined the evolutionary interdependence of leaf flammability with leaf functional and life‐history traits related to plant strategies in fire‐prone vegetation. Here, we determined evolutionary correlations between leaf flammability and leaf water content, leaf mass per area, leaf area, plant growth form, and fire response strategy. Consideration of leaf flammability covariation with other traits throughout the species’ phylogeny sheds light on a potential nexus of traits that has evolved with leaf flammability through time. Given that we are witnessing a shift toward more frequent high‐severity and high‐intensity fires in many parts of the world (Duane et al., 2021; Cunningham et al., 2024), our research on the evolution of live‐leaf flammability will expand knowledge of how species affect wildfire dynamics and will contribute to the protection of lives, property and biodiversity in fire‐prone systems.

## MATERIALS AND METHODS

### Study species and trait data

We explored the macroevolution of leaf flammability in 75 angiosperm plant species, spanning 34 taxonomic families and five growth forms, that are widespread and abundant in fire‐prone dry sclerophyll forest, one of the most dominant vegetation assemblages in eastern Australia. Three of us (L.K.H, M.L.M. [as M.L.P.], and B.R.M.) did the original field sampling, flammability experiments, and laboratory measurements of samples, and methodological details and information on the species and their traits were described by Murray et al. (2013).

Briefly, leaves were collected in dry sclerophyll forest sites in the Greater Sydney Region, which has a mean minimum and maximum temperature of 13.8°C and 21.8°C, respectively, and a mean annual rainfall of 1211.1 mm (Australian Bureau of Meteorology, 2024). Leaf flammability was measured as the time taken (s) for an individual live leaf to ignite after being exposed to an ignition source (i.e., leaf ignitability; Anderson, 1970) in a muffle furnace using of Gill and Moore (1996) and De Lillis et al. (2009) as adapted and described by Murray et al. (2013). Leaf area (LA) was measured as a one‐sided projected area by scanning leaves on a flatbed scanner and analysing scans in ImageJ (Schneider et al., 2012). Fresh leaves were weighed on a scientific balance to determine leaf fresh mass (LFM). Leaves were then dried for 48 h at 75°C and weighed again to obtain the leaf dry mass (LDM). Leaf mass per area (LMA) was calculated as the ratio of leaf dry mass (LDM) to leaf area (LA), representing the biomass per unit of leaf area required to reach ignition under heat. Percent leaf water content (LWC) was calculated as 100 × [(LFM – LDM)/LFM], representing the proportion of water (relative to a leaf's fresh mass) that must be vaporized before ignition.

We obtained data for each species on their growth form and fire response from published sources. The growth form of each species was classified as either climber, herb, shrub, shrub/tree, or tree PlantNET, 2024). The fire response of each species was classified in relation to post‐fire resprouting capacity based on a curated plant trait database for the Australian flora (Falster et al., 2021). Species were assigned to one of three categories according to resprouting capacity after a wildfire that causes 100% leaf scorch: (1) fire‐sensitive (species with fewer than 30% of plants resprouting), (2) fire‐tolerant (species with more than 70% of plants resprouting), or (3) weak resprouters (species with between 30% and 70% of plants resprouting).

### Phylogenetic tree

**Figure 1:**
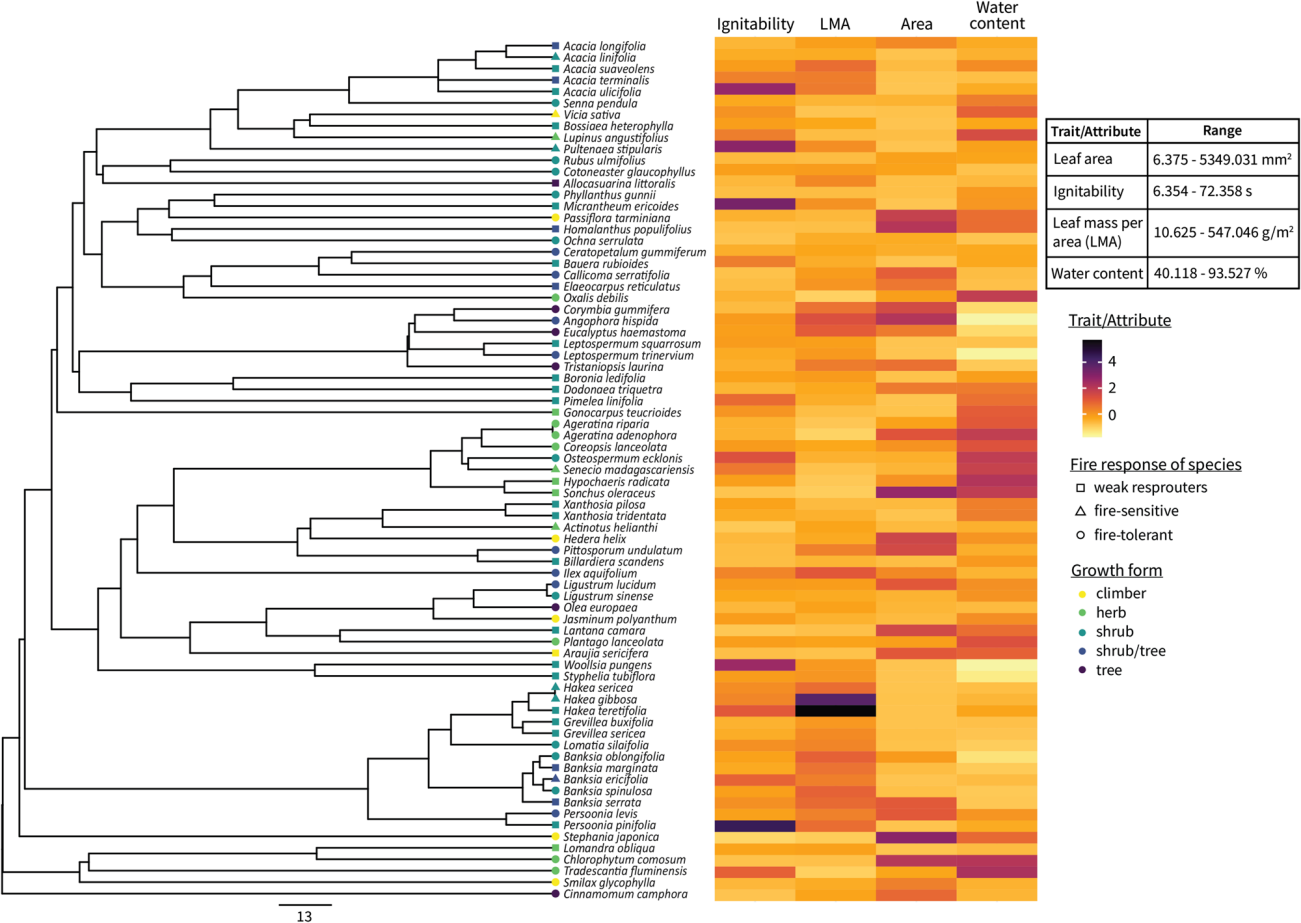
Time‐calibrated phylogeny of the 75 plant species of fire‐prone dry sclerophyll forest. Log‐transformed values of leaf ignitability, leaf mass per area (LMA), leaf area (LA), and leaf water content (LWC) have been scaled for comparison in the heatmap. The scale underneath the phylogeny represents millions of years.

Phylogenetic comparative methods require the construction of a phylogenetic tree of the study species to combine with trait data. We used a mega‐tree approach to generate a time‐calibrated phylogenetic tree (Figure 1). The phylogeny was assembled using the R package V.Phylomaker2 (Jin and Qian, 2022) and R version 4.4.1 (R Core Team, 2024). Mega‐tree data were derived from the largest dated phylogeny for seed plants developed by Smith and Brown (2018) based on molecular data from GenBank and data from the Open Tree of Life project. In the tree, 15 species were resolved to genus level and two species to family level. In two instances, species synonyms validated by PlantNET (2024) were used to resolve their tree position (*Leptospermum trinervium* and *Phyllanthus gunnii*). The time‐calibrated phylogenetic tree file is presented in Appendix S1.

### Phylogenetic signal

We estimated phylogenetic signal on log‐transformed data for leaf flammability using the R packages ape (v5.8; Paradis and Schliep, 2019) and phytools (v2.3‐0; Revell, 2012). We used Blomberg's *K* (Blomberg et al., 2003), where *K* ranges from 0 to >1, and Pagel's *λ* (Pagel, 1999), where *λ* typically ranges between 0 and 1. Values of Blomberg's *K* > 1 and Pagel's *λ* close to 1 indicate that closely related species are more similar in leaf flammability than expected under a Brownian motion (random walk) model of trait evolution, suggesting strong phylogenetic signal. In contrast, *K* < 1 and *λ* close to 0 indicate that closely related species are less similar in leaf flammability than expected under Brownian motion, suggesting a weak phylogenetic signal.

### Evolutionary models of leaf flammability

Central to phylogenetic comparative methods are mathematical models that connect trait phenotypes to the branching pattern of a phylogeny (Cressler et al., 2015). We built models of leaf flammability evolution with maximum likelihood methods following procedures described by Revell and Harmon (2022). First, we used the R package geiger (v2.0.11; Harmon et al., 2008; Pennell et al., 2014) to fit and compare six models of trait evolution including pure BM; Pagel's lambda (*λ*), kappa (*κ*), and delta (*δ*); an OU process with a single optimum; and a white‐noise (WN) process. We then fitted two different OU multiple optima (OUM) models using the R package OUwie (v2.10; Beaulieu and O'Meara, 2012). We used Akaike's information criterion corrected for small sample size (AICc) to compare the fit of our models. The best fitting models were selected as those with the lowest AICc and the largest Akaike weight.

The pure BM model is a neutral model of evolution, where traits evolve under random genetic drift and without any stabilizing selection (Cavalli‐Sforza and Edwards, 1967). We applied the BM model here to test whether changes in leaf flammability across the phylogeny have accumulated in a manner directly proportional to the branch lengths of the phylogenetic tree. Pagel's three models of evolution are based on BM, but involve transformations applied to branch lengths of the phylogenetic tree (Pagel, 1999). In the *λ* model, a value of *λ* = 1 corresponds to a true BM model of leaf flammability evolution, with values of *λ* decreasing from 1 toward 0 indicating that phylogenetic history has diminishing effects for predicting leaf flammability. Pagel's *κ* tests the tempo of evolution, and we used it to seek evidence for either a punctuational or gradual mode of leaf flammability evolution. We used Pagel's *δ* to detect whether the rate of leaf flammability evolution has accelerated or slowed over time from the root to the tips of the phylogeny. Values of *δ* < 1 indicate rapid, early (i.e., adaptive) evolution of leaf flammability, with slower rates of change among closely related species. Values of *δ* > 1 provide a signature of accelerating evolution as time progresses, marked by faster rates of change among closely related species. We also considered a null model of phylogenetically independent evolution, the white‐noise (WN) model, that ignores phylogenetic relatedness across species as if they were placed at equal distance on a star phylogeny (Flores et al., 2014). This model serves as an important null hypothesis that assumes an absence of phylogenetic covariance in leaf flammability.

We built an OU model of leaf flammability evolution that assumes a trait evolves towards an optimal value (*θ*), such that deviations from this optimum undergo stabilizing selection to revert to the optimal value (Hansen, 1997). We also applied an extension of the OU model, the OUM model, that allows for different optimal values for different regimes in the phylogeny (Butler and King, 2004; Beaulieu et al., 2012). Here, we explored the potential for different values of θ for leaf flammability along two separate evolutionary regimes. We built one OUM model that specified growth form as a selective regime, and a second OUM model that specified fire response as a selective regime, based on previous findings that flammability can vary as a function of growth form (Mason et al., 2016; Cui et al., 2020b; Potts et al., 2022) and fire response strategy (Michelaki et al., 2020).

### Ancestral state reconstruction

The OUM models required ancestral state reconstruction of growth form and fire response based on the phylogeny. The method used for ancestral state reconstruction must be selected carefully to ensure accuracy and reliability of inferred ancestral traits (Hardy, 2006; Revell, 2025). To enhance the reliability and accuracy of our ancestral state reconstruction, we used stochastic character mapping (SCM) in the R package phytools (v2.3‐0; Revell, 2012) to estimate ancestral states. The process of character evolution is modelled in SCM in a probabilistic manner, which is particularly useful when reconstructing ancestral states for discrete traits. In addition, SCM considers varying rates of evolution across different branches of the tree, which is important for realistic modeling of character evolution. We fitted three simple models of evolution to determine how each growth form and fire response transitioned throughout the phylogenetic tree. These were the equal rates (ER), symmetric backward and forward rates (SYM), and all‐rates‐different (ARD) models. Using AICc and AICc weights, which considered uncertainty in the estimation of ancestral states (Maccagni and Willi, 2022), we determined that the best‐fitting models for growth form and fire response were the SYM and ARD models, respectively (Appendix S2). Along branches of the tree, the SYM model indicates that rates of transition between different growth forms have been symmetrical, while the ARD model allows for different rates of transition between fire response categories. For each of growth form and fire response, we generated 1000 stochastic character maps based on the ARD model, to model discrete character evolution on the reconstructed phylogeny (Revell and Harmon, 2022). We used the method best within the function ace, which uses maximum likelihood to estimate the most likely ancestral states based in the 1000 character maps.

### Evolutionary correlations between leaf flammability and plant traits

We implemented a phylogenetic generalized least squares (PGLS) analysis (Grafen, 1989; Martins and Hansen, 1997) to quantify evolutionary correlations between leaf flammability (response variable) and LWC, LMA, LA, plant growth form, and fire response strategy (explanatory variables). The traits LWC, LMA, and LA were all log‐transformed before analysis. We built a PGLS multiple regression model using the R package NLME (Pinheiro et al., 2021) and used a marginal model approach to determine the significant and unique contribution of each trait to variation in leaf flammability. This test evaluates the significance of each explanatory variable by comparing the full model (with all explanatory variables) to a reduced model (excluding the explanatory variable of interest). Regression parameters and the best‐fitting value of Pagel's *λ* for the covariance matrix of the residuals were estimated via maximum likelihood. The *λ* estimated for the covariance matrix in the PGLS is not the phylogenetic signal *λ* of the single leaf flammability trait involved in tests of phylogenetic signal (Pearse et al., 2023).

## RESULTS

**Figure 2:**
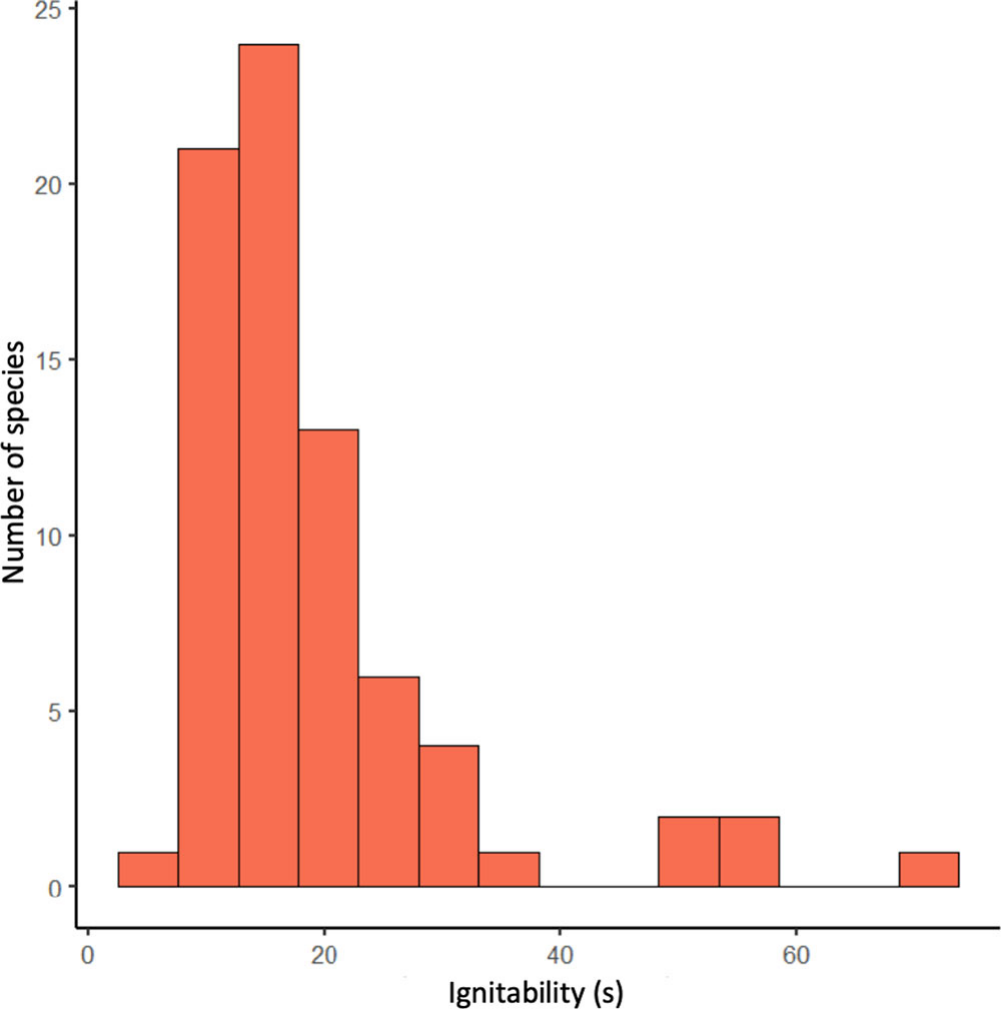
Frequency histogram of untransformed leaf ignitability values across the 75 study species.

Live‐leaf flammability assessed as leaf ignitability varied across species from 6.4 s (*Stephania japonica*) to 72.4 s (*Persoonia pinifolia*) and showed a right‐skewed distribution (mean = 19.6 s ± 11.9 SD, median = 16.4 s, interquartile range = 8.5 s; Figure 2). There was no significant phylogenetic signal in leaf flammability based on Pagel's *λ* (*λ* = 0.16, *P* = 0.34). Significant but weak signal was found with Blomberg's *K* (*K* = 0.32, *P* = 0.005). Phenotypic evolution of leaf flammability was best fitted equally by the OU model (*α* = 0.05, *σ* ^2^ = 0.02, *t*_1/2_ = 14.1, *θ* = 16.6 s ± 1.1 SE) and by Pagel's *δ* model (*δ* = 13.4, *σ* ^2^ = 0.002). Both the OU and *δ* models produced similarly low AICc values and weights (Table 1). In the PGLS multiple regression model, significant and unique evolutionary correlations were found between leaf flammability and the three leaf traits, with fast ignition times (i.e., high flammability) correlated with low LWC (*F* = 11.8, df = 1, *P* = 0.001), low LMA (*F* = 16.6, df = 1, *P* = 0.0001), and large LA (*F* = 27.7, df = 1, *P* < 0.0001). There were no significant evolutionary correlations between leaf flammability and either fire response (*F* = 0.6, df = 2, *P* = 0.6) or growth form (*F* = 0.4, df = 4, *P* = 0.8).

**Table 1:** Results of modelling leaf flammability evolution using Brownian motion (BM), Pagel's *λ*, *κ*, and *δ*, nonphylogenetic white noise (WN), Ornstein–Uhlenbeck (OU), and Ornstein–Uhlenbeck multiregime (OUM) approaches. The OUM (fire) and OUM (growth) models were used to examine fire response and growth form as selective regimes on leaf flammability evolution. The AICc weights for comparisons among the first six models were obtained using the R package Geiger, and the AICc weights for comparison between the OU, OUM (fire), and OUM (growth) models were obtained using the R package OUwie.

Model	Log‐likelihood	AICc	AICc weight (geiger)	AICc weight (OUwie)
BM	–60.7	125.6	5.0 × 10^–7^	
*λ*	–48.5	103.4	3.1 × 10^–2^	
*κ*	–54.3	114.9	1.1 × 10^–4^	
*δ*	–45.9	98.2	4.5 × 10^–1^	
WN	–49.0	102.2	6.2 × 10^–2^	
OU	–45.9	98.2	4.5 × 10^–1^	(a) 7.5 × 10^–1^ (b) 9.2 × 10^–1^
OUM (fire)	–44.7	100.4		(a) 2.5 × 10^–1^
OUM (growth)	–43.7	103.0		(b) 8.2 × 10^–2^

*Notes*: AICc weights for the OU and OUM models are dependent on the model under comparison. In the table in the far‐right column, (a) shows the comparison of weights between the OU model and the OUM model under the fire response regime, and (b) shows the comparison of weights between the OU model and the OUM model under the growth form regime.

## DISCUSSION

Our study provides evidence that the evolution of live‐leaf flammability has not followed a pure BM process in plant species of fire‐prone dry sclerophyll forests in eastern Australia. We found that the pure BM model emerged as the weakest fitting macroevolutionary model. We found no evidence of strong phylogenetic structure in leaf flammability based on either Pagel's *λ* or Blomberg's *K*, demonstrating that leaf flammability varies more widely among related species than expected under strong phylogenetic conservatism. For instance, within the genus *Acacia*, ignition times ranged from 12.4 s in *A. longifolia* to 51.2 s in *A. ulicifolia*. Similarly, at the family level, ignition times in the Proteaceae, ranged from 13.2 s in *Grevillea buxifolia* to 72.4 s in *Persoonia pinifolia*. These findings suggest that leaf flammability is an evolutionarily labile trait, unlike other plant traits that show strong phylogenetic signal (e.g., plant height, Portelli et al., 2023; leaf area, Wang et al., 2023; seed mass, Carta et al., 2022; sapwood density, Liu et al., 2015; and LMA, Flores et al., 2014).

One of the two best‐fitting evolutionary models of leaf flammability was the OU model, describing stabilizing selection toward an optimal ignitability value (*θ* = 16.6 s). The OU model is a modified BM model with a mean‐reverting property (Hansen, 1997), such that extreme trait values are selected against. The emergence of the OU model as a good fit for leaf flammability is consistent with the weak level of phylogenetic signal detected by Blomberg's *K*. Both pieces of evidence imply that evolutionary processes have influenced leaf flammability to some extent, but other ecological or environmental factors may also play a role in shaping flammability. To critically understand the dynamics of the OU model (Grabowski et al., 2023), we need to carefully interpret the three model parameter estimates: *α* (which describes the strength of selection's pull on a trait back to the optimal value), *t*_1/2_ (which describes the evolutionary half‐life, or the average time for a trait to evolve halfway from an ancestral state toward a new optimum), and *σ* ^2^ (which describes the amount of random drift influencing a trait). Our OU model for leaf flammability had a low value of *α* (0.05), a high value of *t*_1/2_ (14.1), and a low value of *σ* ^2^ (0.02). Together, these estimates indicate that leaf flammability is under a weak reversionary force toward the optimum (*α*), with deviations from the optimum reversed gradually over evolutionary time (*t*_1/2_), and a weak influence of random drift (*σ* ^2^). The emergence of an optimal value of leaf ignitability could reflect an evolutionary trade‐off, with on the one hand, selective pressure for increased flammability and on the other hand, selective pressure to avoid excessive damage from fire.

We describe two possible explanations for the nature of the fit of the OU model to leaf flammability evolution. First, even in the presence of direct selection for an optimum flammability value, stronger selective pressures on other leaf traits could provide antagonistic forces, leading to a weak reversionary force toward the optimum flammability value. For instance, the evolution of leaf traits in response to environmental conditions such as water availability (LWC), light harvesting (LA), and herbivore defense (LMA) could provide such antagonistic forces because they likely take primacy for plant fitness. In this context, strong selective pressures for low LWC in some species and high LWC in others would act to weaken the pull toward an optimum value of leaf flammability given the observed relationships between LWC and leaf flammability. Similar arguments could be made for differential selection for high vs low LMA and small vs large LA among species providing antagonistic forces on leaf flammability evolution. A second explanation for the emergence of weak stabilizing selection on leaf flammability is that the opposing selective forces on LWC, LMA, and LA suggested above could lead to the appearance of stabilizing selection in an OU model, when in fact, there is no direct selection on leaf flammability. In other words, opposing selective pressures on other leaf traits mimic stabilizing selection when none exists. Further work is required to tease apart these two competing explanations of leaf flammability evolution. However, whether direct selection is or is not operating on leaf flammability as offered by these two explanations, the key finding that emerges is that LWC, LMA, and LA are highly important in shaping interspecific variation in leaf flammability.

We found that Pagel's *δ* was an equally well‐fitting model of leaf flammability evolution. In our model, *δ* was high (13.4), demonstrating accelerating rates of evolution of leaf flammability over time (Pagel, 2002). Such rate acceleration raises the question, what are the potential evolutionary pressures on leaf flammability that could have led to higher rates of evolution in more recent times? This issue is complicated, likely related to changes in fire conditions and regimes over evolutionary time, and we do not at present have enough information to answer this question. What is required is a study like that of He et al. (2011), who combined highly calibrated records from the fossil record on speciation events with historical information on climate variation to reconstruct the macroevolution of fire‐related traits in relation to fire conditions. Such a study has the potential to be highly informative, particularly if rates of leaf flammability evolution are found to change in relation to changes in climate and fire conditions, perhaps with leaves evolving toward higher flammability, and especially given global increases in megafires across the world in the Anthropocene.

More broadly, there is a need for further macroevolutionary studies of plant flammability to better understand an increasingly flammable world. While our study focused on live‐leaf flammability, because it is a trait of central importance in flammability research and applied modelling (Zylstra et al., 2016; Tumino et al., 2019; Cawson et al., 2023; Younes et al., 2024), future studies could explore macroevolutionary patterns in other dimensions of live‐leaf and leaf‐litter flammability such as sustainability (the duration of leaf burn), combustibility (how well a leaf burns), and consumability (how much of a leaf is burned), and relationships between these dimensions of flammability and leaf morphological and chemical traits in a phylogenetic context (e.g., Engber and Varner, 2012; Mason et al., 2016; Simpson et al., 2016; Varner et al., 2022). These would also be important matters for investigation not just at the leaf level but also at the shoot level (e.g., Cui et al., 2020a, 2020b; Murray et al., 2023; Budha‐Magar et al., 2024; Alam et al., 2025). In addition, future studies could explore the evolution of leaf and shoot flammability in relation to other fire‐related traits such as branch self‐pruning, bark thickness, seed‐banking and cone serotiny.

Our study focused on plant species of fire‐prone dry sclerophyll forests of eastern Australia. More work is needed for a wide range of fire‐prone and non‐fire‐prone vegetation assemblages in Australia and across the rest of the world to determine the generality of our findings. In particular, comparisons with leaf flammability macroevolution on other continents will help to determine whether Australia's long geographic isolation might have played a role in generating idiosyncratic fire ecology patterns on the continent. What prompts this thinking is that Varner et al. (2022) found evidence of phylogenetic signal in leaf‐litter flammability among 31 *Pinus* species of the northern hemisphere. Within Australia, it will be important to distinguish idiosyncratic from general patterns in leaf flammability evolution between fire‐prone and non‐fire‐prone vegetation assemblages and among plant assemblages such as wet sclerophyll forest, rainforest, heathland, and semiarid woodlands for instance. From a mechanistic perspective, it would be helpful to know if low LWC, low LMA, and large LA are significant drivers of interspecific variation in leaf flammability in these plant assemblages. Finally, it will also be interesting to target genera containing species that occur in vegetation assemblages which vary in proneness to fire (e.g. Clarke et al., 2014) to begin to assess the role of fire history on leaf flammability patterns in an evolutionary context.

Overall, our study provides some tantalizing evidence that leaf flammability may be a trait under stabilizing selection (Pan et al., 2014; Cui et al., 2020a), albeit of a weak nature. There has been much discussion about whether plants should or should not evolve to be flammable (Mutch, 1970; Bond and Midgley, 1995; Schwilk and Ackerly, 2001; He et al., 2011). Our findings in the present study contribute to this body of work and suggest fruitful areas for future investigation about macroevolutionary patterns in plant flammability.

## AUTHOR CONTRIBUTIONS

B.R.M. and L.K.H. conceived the initial project. L.K.H., M.L.M., and B.R.M. collected the original data and did the flammability experiments and measured leaf samples. B.R.M, Z.A.X., and M.L.M. performed the macroevolutionary analyses. Z.A.X. and M.L.M. produced the figures. All authors contributed to writing the manuscript.

## Supporting information


**Appendix S1.** The time‐calibrated phylogenetic tree file used in our comparative analyses.

**Appendix S2.** Results of model comparisons between the equal rates (ER), symmetric backward and forward rates (SYM), and all‐rates‐different (ARD) models using AICc and AICc weights for (a) growth form and (b) fire response.

## Data Availability

Trait data were sourced from Murray et al. (2013), PlantNET (2024), and Falster et al. (2021). The time‐calibrated phylogenetic tree file used in our analyses is available in Appendix S1.
