# Supplementary material for: Toward a macroevolutionary understanding of live‐leaf flammability in plant species of fire‐prone forests
Source: Am J Bot. 2025 Jul 10;112(10):e70073. doi: 10.1002/ajb2.70073 (PMC12572687; doi:10.1002/ajb2.70073)
Supplement: Supplementary file 1 — Appendix S1. The time‐calibrated phylogenetic tree file used in our comparative analyses. [file AJB2-112-e70073-s002.docx]

**Appendix S1.** The time-calibrated phylogenetic tree file used in our comparative analyses.

(Cinnamomum_camphora:135.912187,((Smilax_glycophylla:116.876807,(Tradescantia_fluminensis:114.600563,(Chlorophytum_comosum:58.679716,Lomandra_obliqua:58.679716)N59333:55.920847)mrcaott121ott334:2.276244)mrcaott121ott1439:18.881258,(Stephania_japonica:131.6807,(((Persoonia_pinifolia:12.115705,Persoonia_levis:12.115705)N52755:33.938567,((Lomatia_silaifolia:25.678486,(((Hakea_gibbosa:0.142338,Hakea_sericea:0.142338)N52363:6.463905,Hakea_teretifolia:6.606242)N52339:4.001208,(Grevillea_sericea:8.054218,Grevillea_buxifolia:8.054218)N52266:2.553233)N52224:15.071035)N52220:5.507827,(((Banksia_spinulosa:3.00953,Banksia_ericifolia:3.00953)N52117:2.511942,(Banksia_marginata:3.8799,Banksia_oblongifolia:3.8799)N52103:1.641572)N52102:2.548999,Banksia_serrata:8.070471)N51978:23.115842)N51973:14.86796)N51970:84.270258,((Gonocarpus_teucrioides:122.405092,(((Tristaniopsis_laurina:36.373487,((Leptospermum_trinervium:17.580973,Leptospermum_squarrosum:17.580972)N48972:18.412419,((Angophora_hispida:24.90782,Corymbia_gummifera:24.90782)N48835:9.630587,Eucalyptus_haemastoma:34.538407)N48324:1.454985)N48320:0.380095)N48319:80.619709,((Dodonaea_triquetra:79.148401,Boronia_ledifolia:79.148401)N45722:25.109657,Pimelea_linifolia:104.258058)mrcaott96ott378:12.735137)mrcaott96ott607:1.585408,(((Oxalis_debilis:91.346395,(((Bauera_rubioides:50.08571,Ceratopetalum_gummiferum:50.08571)N42619:8.042609,Callicoma_serratifolia:58.128319)N42538:26.399993,Elaeocarpus_reticulatus:84.528312)N42361:6.818083)N42360:20.080032,((Ochna_serrulata:94.206637,Homalanthus_populifolius:94.206637)N40288:8.401122,((Micrantheum_ericoides:83.778522,Phyllanthus_gunnii:83.778521)N39772:11.133064,Passiflora_tarminiana:94.911586)N37851:7.696173)N37848:8.818668)mrcaott2ott345:4.359138,((Allocasuarina_littoralis:111.148008,(Cotoneaster_glaucophyllus:94.56118,Rubus_ulmifolius:94.56118)N34055:16.586828)mrcaott371ott2511:1.553188,(((Senna_pendula:50.699848,((Acacia_suaveolens:19.580171,(Acacia_linifolia:12.134539,Acacia_longifolia:12.134539)N32443:7.445632)N32437:9.238128,Acacia_ulicifolia:28.818298,Acacia_terminalis:28.818298)N32426:21.881549)N32416:27.263339,(Lupinus_angustifolius:64.215926,(Bossiaea_heterophylla:60.311475,Vicia_sativa:60.311475)N29712:3.904451)N29709:13.747261)N29707:6.800151,Pultenaea_stipularis:84.763337)N29704:27.937858)mrcaott371ott579:3.084369)mrcaott2ott371:2.793039)mrcaott2ott96:3.826488)mrcaott2ott2464:1.329145,((Styphelia_tubiflora:59.100994,Woollsia_pungens:59.100993)N21563:53.239735,((Araujia_sericifera:89.751068,((Jasminum_polyanthum:29.989738,((Ligustrum_sinense:2.129923,Ligustrum_lucidum:2.129923)N16047:17.981658,Olea_europaea:20.111581)N15925:9.878158)N15924:41.03087,(Plantago_lanceolata:52.932762,Lantana_camara:52.932762)N11378:18.087847)N11375:18.730459)mrcaott248ott1191:16.990343,(Ilex_aquifolium:102.692841,(((Billardiera_scandens:19.120713,Pittosporum_undulatum:19.120713)N10434:44.248486,(Hedera_helix:60.188166,(Actinotus_helianthi:42.464986,(Xanthosia_tridentata:12.268866,Xanthosia_pilosa:12.268866)N09877:30.19612)N09863:17.72318)N07809:3.181033)N07808:30.361013,((Sonchus_oleraceus:12.541262,Hypochaeris_radicata:12.541262)N04759:18.132877,((Senecio_madagascariensis:21.448779,Osteospermum_ecklonis:21.448778)N02470:1.540681,(Coreopsis_lanceolata:18.158008,(Ageratina_adenophora:0.714585,Ageratina_riparia:0.714585)N00287:17.443423)N00019:4.831452)N00014:7.684679)N00012:63.056073)mrcaott320ott1673:8.962629)campanulids:4.04857)mrcaott248ott320:5.599318)mrcaott248ott650:11.393508)Pentapetalae:6.590294)mrcaott2ott969:1.356169)eudicotyledons:4.077365)mrcaott2ott121:0.154122)Mesangiospermae:1.781936;
