# Supplementary material for: Toward a macroevolutionary understanding of live‐leaf flammability in plant species of fire‐prone forests
Source: Am J Bot. 2025 Jul 10;112(10):e70073. doi: 10.1002/ajb2.70073 (PMC12572687; doi:10.1002/ajb2.70073)
Supplement: Supplementary file 2 — Appendix S2. Results of model comparisons between the equal rates (ER), symmetric backward and forward rates (SYM), and all‐rates‐different (ARD) models using AICc and AICc weights for (a) growth form and (b) fire response. [file AJB2-112-e70073-s001.docx]

**Appendix S2.** Results of model comparisons between the equal rates (ER), symmetric backward and forward rates (SYM), and all-rates-different (ARD) models using AICc and AICc weights for (a) growth form and (b) fire response.

| Model | Log-likelihood | AICc | AICc weight |
| --- | --- | --- | --- |
| (a) Growth form |  |  |  |
| SYM | -98.5 | 220.4 | 1.0 |
| ER | -112.9 | 227.9 | 0.02 |
| ARD | -92.7 | 241.0 | 0.0 |
| (b) Fire response |  |  |  |
| ARD | -69.8 | 152.9 | 1.0 |
| ER | -80.4 | 162.8 | 0.007 |
| SYM | -80.3 | 167.0 | 0.001 |
